# Discovery and genome characterization of three new Jeilongviruses, a lineage of paramyxoviruses characterized by their unique membrane proteins

**DOI:** 10.1186/s12864-018-4995-0

**Published:** 2018-08-16

**Authors:** Bert Vanmechelen, Magda Bletsa, Lies Laenen, Ana Rita Lopes, Valentijn Vergote, Leen Beller, Ward Deboutte, Miša Korva, Tatjana Avšič Županc, Joëlle Goüy de Bellocq, Sophie Gryseels, Herwig Leirs, Philippe Lemey, Bram Vrancken, Piet Maes

**Affiliations:** 1 Department of Microbiology and Immunology, Laboratory of Clinical Virology, Rega Institute for Medical Research, KU Leuven – University of Leuven, Herestraat 49, Box 1040, BE3000 Leuven, Belgium; 2 Department of Microbiology and Immunology, Laboratory of Evolutionary and Computational Virology, Rega Institute for Medical Research, KU Leuven – University of Leuven, Herestraat 49, Box 1040, BE3000 Leuven, Belgium; 3 Department of Microbiology and Immunology, Laboratory of Viral Metagenomics, Rega Institute for Medical Research, KU Leuven – University of Leuven, Herestraat 49, Box 1040, BE3000 Leuven, Belgium; 4 Institute of Microbiology and Immunology, Faculty of Medicine, University of Ljubljana, Zaloška 4, SI-1000 Ljubljana, Slovenia; 5 The Czech Academy of Sciences, Institute of Vertebrate Biology, Květná 8, 603 65 Brno, Czech Republic; 6 Department of Biology, Evolutionary Ecology Group, University of Antwerp, Universiteitsplein 1, 2610 Antwerpen, Belgium; 7 Ecology and Evolutionary Biology Department, University of Arizona, 1041 E. Lowell St, Tucson, AZ 85719 USA

**Keywords:** PMPV-1, MMLPV-1, MMLPV-2, G protein, Cell attachment protein, Rodent paramyxovirus

## Abstract

**Background:**

In the past decade, many new paramyxoviruses that do not belong to any of the seven established genera in the family *Paramyxoviridae* have been discovered. Amongst them are J-virus (JPV), Beilong virus (BeiPV) and Tailam virus (TlmPV), three paramyxovirus species found in rodents. Based on their similarities, it has been suggested that these viruses should compose a new genus, tentatively called ‘Jeilongvirus’.

**Results:**

Here we present the complete genomes of three newly discovered paramyxoviruses, one found in a bank vole (*Myodes glareolus*) from Slovenia and two in a single, co-infected Rungwe brush-furred rat (*Lophuromys machangui*) from Mozambique, that represent three new, separate species within the putative genus ‘Jeilongvirus’. The genome organization of these viruses is similar to other paramyxoviruses, but like JPV, BeiPV and TlmPV, they possess an additional open reading frame, encoding a transmembrane protein, that is located between the F and G genes. As is the case for all Jeilongviruses, the G genes of the viruses described here are unusually large, and their encoded proteins are characterized by a remarkable amino acid composition pattern that is not seen in other paramyxoviruses, but resembles certain motifs found in *Orthopneumovirus* G proteins.

**Conclusions:**

The phylogenetic clustering of JPV, BeiPV and TlmPV with the viruses described here, as well as their shared features that set them apart from other paramyxoviruses, provide additional support for the recognition of the genus ‘Jeilongvirus’.

**Electronic supplementary material:**

The online version of this article (10.1186/s12864-018-4995-0) contains supplementary material, which is available to authorized users.

## Background

Paramyxoviruses constitute a family (*Paramyxoviridae*) of enveloped, negative-sense single-stranded RNA viruses that infect a wide range of natural hosts, from reptiles, birds and fish to a variety of mammals, including humans [1]. Paramyxoviruses have been implicated as the causative agent of several human diseases, including mumps (mumps virus), measles (measles virus) and a collection of respiratory tract infections (human parainfluenza viruses) [2–4]. In addition to human disease, paramyxoviruses are also associated with multiple animal pathologies and some, including the highly pathogenic Hendra virus and Nipah virus, are known to be zoonotic pathogens [5]. All members of the family *Paramyxoviridae* share a similar genome organization, with a single 15–21 kb RNA segment containing 6–9 viral genes. As is typical for members of the order *Mononegavirales*, the gene sequence within each genome is the same for all paramyxoviruses, with genes located at the 3′ end being transcribed in greater abundance than those at the 5′ end [6].

The family *Paramyxoviridae* currently contains 55 recognized species, organized in seven genera (*Aquaparamyxovirus*, *Avulavirus*, *Ferlavirus*, *Henipavirus*, *Morbillivirus*, *Respirovirus* and *Rubulavirus*) and 15 putative species that are yet to be classified [7]. A significant number of these putative species does not seem to belong to any of the seven existing paramyxovirus genera. These include four recently discovered fish paramyxoviruses, Salem virus, isolated from a horse (*Equus caballus*), Tupaia paramyxovirus, discovered in a tree shrew (*Tupaia belangeri*) and bank vole virus (BaVV), Beilong virus (BeiPV), Tailam virus (TlmPV), J-virus (JPV), rodent paramyxovirus (Rodent PV), Mossman virus and Nariva virus, all discovered in rodents [8–16]. There are currently no uniform standards for the delimitation of genera in the family *Paramyxoviridae*, but because of their sequence similarity and unique genome structure, it has been proposed that BeiPV, TlmPV and JPV should be classified together to form a new genus, provisionally named ‘Jeilongvirus’ [10, 17]. The yet undescribed ‘rodent paramyxovirus’ (GenBank: KY370098) most likely also belongs to this putative genus.

Here we report the complete genome sequence of three novel rodent paramyxoviruses, thought to represent three new species within the family *Paramyxoviridae*. These viruses were detected via next-generation sequencing of rodent samples previously found to be positive for hantavirus or hepacivirus. One virus was found in the kidney and lung of a bank vole (*Myodes glareolus*), captured in Slovenia. The other two viruses were discovered in the kidney of a Rungwe brush-furred rat (*Lophuromys machangui*), captured in Mozambique. Referring to their respective hosts and the locations in which these hosts were caught, the three viruses described here were given the names Mount Mabu Lophuromys paramyxovirus 1 (MMLPV-1), Mount Mabu Lophuromys paramyxovirus 2 (MMLPV-2) and Pohorje Myodes paramyxovirus 1 (PMPV-1). All three viruses cluster phylogenetically with JPV, TlmPV, BeiPV and Rodent PV, providing additional support for the recognition of the proposed genus ‘Jeilongvirus’ as a separate genus within the family *Paramyxoviridae*.

## Results

### Discovery of MMLPV-1, MMLPV-2 and PMPV-1

**Fig. 1:**
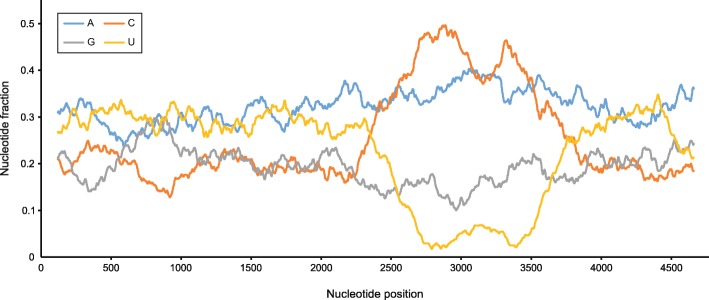
Nucleotide composition of the PMPV-1 G gene. A sliding window view of the nucleotide composition of the PMPV-1 G gene, using 5% of the gene length as window size, shows that this gene has a low GC-content, comparable to the rest of the PMPV-1 genome, as well as a ~ 1400 nt-long region that is marked by a near-absolute absence of uracils (< 5%) and an abundance of cytosines (up to 50%). The unique nucleotide composition of the PMPV-1 G gene is also reflected in the amino acid composition of the corresponding G protein (see Fig. 5)

In this study, we determined the complete genome sequences of three putative new paramyxovirus species. Two sequences were assembled from Illumina sequencing data of RNA extract obtained from a kidney of a Rungwe brush-furred rat, trapped on Mount Mabu, Mozambique. The third sequence originated from viral RNA extracted from a lung and kidney of a bank vole that was caught in Slovenia. Despite the high overall coverage of Illumina data, reads spanning a specific part of the G ORF of this third sequence appeared to be absent. Sequence analysis of this region, following RT-PCR amplification and Sanger sequencing, revealed an unusual nucleotide composition (C-rich and T-poor), presumably resulting in the formation of RNA secondary structures that hinder efficient reverse transcription and thus Illumina sequencing (Fig. 1). The complete genomes of the paramyxoviruses described here have GC-contents of 37.1, 37.0 and 40.9%, and are 17,940, 17,220 and 20,148 nucleotides long, respectively, implying all three genomes adhere to the ‘rule-of-six’. Adherence to this rule, which states that replication of paramyxovirus genomes is only efficient when the genome length is a multiple of six, is a characteristic feature of members of the family *Paramyxoviridae* [18]. The identity of the viruses’ hosts was confirmed by assembling their respective cytochrome b genes from the Illumina data.

### Phylogenetic analysis of MMLPV-1, MMLPV-2 and PMPV-1

**Fig. 2:**
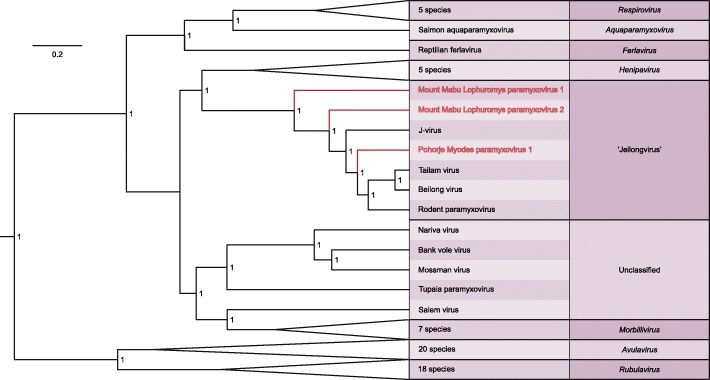
Maximum clade credibility tree of 69 currently known paramyxovirus species. The phylogeny imputed from the concatenated sequence of the N, P, M, F, G and L proteins of 69 known paramyxovirus species shows that MMLPV-1, MMLPV-2 and PMPV-1 (marked in red) cluster with perfect support as separate species together with JPV, BeiPV, TlmPV and rodent PV. Branches of recognized genera that contain multiple species have been collapsed. An expanded version of this tree is given in Additional file 1: Figure S1. A version of this tree based only on the L protein but including the recently discovered fish paramyxoviruses is given in Additional file 2: Figure S2. Branch lengths are scaled and represent the number of amino acid substitutions per site. Numbers at the different nodes indicate the posterior support for a cluster. GenBank accession numbers of all used sequences are provided in Additional file 3: Table S1

As determined by pairwise sequence comparison using the US National Center for Biotechnology Information (NCBI) PAirwise Sequence Comparison (PASC) tool, MMLPV-1 and MMLPV-2 are most similar (44.31% and 52.55%, respectively) to J-virus (NC007454), while PMPV-1 shares the most similarity (51.51%) with Tailam virus (NC025355) [19]. Phylogenetic analysis using Bayesian inference, based on the amino acid sequence of the N, P, M, F, G and L ORFs of all 55 currently recognized, as well as 14 unclassified or putative paramyxovirus species, groups MMLPV-1, MMLPV-2 and PMPV-1 together with, but clearly distinct from, JPV, TlmPV, BeiPV and Rodent PV (Fig. 2; Additional file 1: Figure S1). Due to the difficulties in identifying their ORFs, the four newly discovered fish paramyxoviruses were omitted from this analysis [16]. A separate analysis, based only on the L ORF but including these four viruses, shows a comparable positioning of MMLPV-1, MMLPV-2 and PMPV-1 within the family *Paramyxoviridae* (Additional file 2: Figure S2). Together with JPV, TlmPV, BeiPV and Rodent PV, these viruses appear to constitute a yet unrecognized genus in the family *Paramyxoviridae*, with each virus representing a separate species, something that can also be observed when comparing the proteins of MMLPV-1, MMLPV-2 and PMPV-1 to the proteins of other paramyxoviruses (Table 1).

**Table 1:** Amino acid identities of PMPV-1, MMLPV-1 and MMLPV-2 compared to other paramyxoviruses

		Pohorje Myodes paramyxovirus 1								Mount Mabu Lophuromys paramyxovirus 1							Mount Mabu Lophuromys paramyxovirus 2						
		N	P	M	F	SH^a^	TM^a^	G	L	N	P	M	F	TM^a^	G	L	N	P	M	F	TM^a^	G	L
‘Jeilongvirus’	PMPV-1	100	100	100	100	100	100	100	100	51.7	37.4	66.9	54.7	16.8	40.2	63.0	58.8	40.7	74.7	61.5	27.9	55.9	70.4
	MMLPV-1	51.7	37.4	66.9	54.7	–	16.8	40.2	63.0	100	100	100	100	100	100	100	54.3	34.6	70.8	59.0	20.8	44.7	63.9
	MMLPV-2	58.8	40.7	74.7	61.5	–	27.9	55.9	70.4	54.3	34.6	70.8	59.0	20.8	44.7	63.9	100	100	100	100	100	100	100
	BeiPV	63.1	56.1	77.1	74.5	32.3	34.0	70.8	75.3	53.6	35.0	71.1	52.6	18.8	40.2	62.2	60.0	40.7	77.1	60.3	37.1	53.3	70.6
	TlmPV	63.3	56.1	77.4	73.1	32.3	29.9	70.1	75.2	53.1	34.6	70.2	51.5	21.8	38.9	62.6	61.4	40.7	76.8	59.0	33.0	52.9	71.1
	JPV	53.6	52.8	75.0	69.6	23.1	26.9	57.0	73.1	51.7	35.8	69.9	53.2	18.8	40.6	63.6	59.3	39.0	79.2	62.5	38.6	53.5	71.3
	Rodent PV	64.3	53.3	76.2	72.1	33.8	30.5	70.1	76.4	50.5	34.6	72.6	54.9	18.3	40.4	63.0	63.1	40.2	79.2	62.1	31.5	52.9	71.0
Unclassified	BaVV	40.5	26.4	48.8	31.5	–	–	17.4	52.0	44.0	28.5	53.3	29.8	–	19.1	52.9	44.8	23.6	48.8	33.5	–	18.7	51.2
*Henipavirus*	HeV	37.6	28.0	50.0	36.2	–	–	22.6	48.3	39.5	25.6	52.7	36.4	–	22.4	48.1	40.7	28.0	51.2	36.6	–	24.5	49.2
*Morbillivirus*	MeV	40.0	22.8	44.9	35.0	–	–	17.4	49.0	41.2	27.2	46.1	32.3	–	15.7	48.9	41.0	22.4	47.6	35.0	–	17.0	49.1
*Respirovirus*	SeV	24.3	18.3	38.0	29.2	–	–	28.8	40.4	25.7	16.3	36.7	29.6	–	31.4	39.8	25.5	17.1	37.0	27.7	–	34.2	41.1
*Aquaparamyxovirus*	AsaPV	28.3	21.5	41.3	39.5	–	–	31.4	40.5	29.3	20.7	42.8	37.7	–	30.8	40.5	30.7	18.3	41.0	37.7	–	36.6	41.9
*Ferlavirus*	FDLV	28.1	19.9	40.4	35.2	–	–	36.8	41.2	28.3	19.5	40.4	35.4	–	34.6	41.1	28.8	14.6	41.6	34.8	–	40.6	41.4
*Rubulavirus*	MuV	26.2	20.7	18.7	27.7	–	–	27.5	30.7	28.1	21.1	21.1	27.7	–	25.4	30.9	29.8	19.1	19.0	27.1	–	29.5	30.6
*Avulavirus*	NDV	27.6	18.7	18.7	25.9	–	–	28.0	27.5	25.2	16.7	22.3	25.5	–	25.4	27.4	27.6	16.3	19.6	25.5	–	30.8	28.0

Identity-based comparison of PMPV-1, MMLPV-1 and MMLPV-2 with other Jeilongviruses and a representative species of each of the different paramyxovirus clades indicates that PMPV-1, MMLPV-1 and MMLPV-2 represent three separate species that cluster as part of the tentative genus ‘Jeilongvirus’. Identities are given per protein, expressed as percentages. *HeV* Hendra virus, *MeV* Measles virus, *SeV* Sendai virus, *AsaPV* Atlantic salmon paramyxovirus, *FDLV* Fer-de-lance virus, *MuV* Mumps virus, *NDV* Newcastle disease virus

^a^Not present in all viruses

### Genome organization of MMLPV-1, MMLPV-2 and PMPV-1

Aside from some minor deviations, members of the family *Paramyxoviridae* share a similar genome organization (3’-N-P/V/C-M-F-G-L-5′). The N, M, F, G and L genes all encode one protein while the P gene encodes, in addition to the viral phosphoprotein, some accessory proteins that arise through leaky scanning (C protein) or RNA editing (V/W proteins). This RNA editing occurs through the addition of one or more guanine residues during transcription, following the recognition of a conserved RNA editing site [20]. Although we did not experimentally ascertain the occurrence of this RNA editing in the case of MMLPV-1, MMLPV-2 or PMPV-1, all three genomes do contain a putative RNA editing site (TTAAAAAAGGCA) within their P gene. This sequence matches a conserved motif sequence (YTAAAARRGGCA) found in all members of the genera *Henipavirus* and *Morbillivirus* as well as in JPV, TlmPV, BeiPV and Rodent PV.

**Fig. 3:**
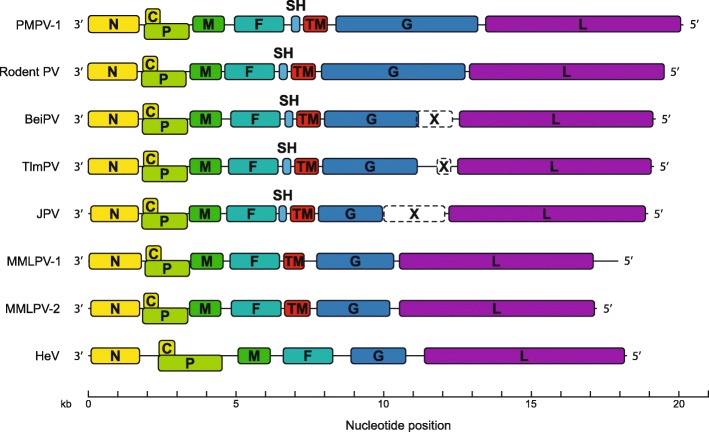
Genome organization of all known ‘Jeilongviruses’. All ORFs are drawn to scale. The X ORF (dashed lines) is found only in the genomes of BeiPV, TlmPV and JPV, and is considered part of the G gene. The SH gene is found only in a limited number of paramyxoviruses, while the TM gene appears to be a unique feature of members of the putative genus ‘Jeilongvirus’. Hendra virus (HeV) is included as a reference non-‘Jeilongvirus’ genome

As indicated by the phylogenetic analysis, MMLPV-1, MMLPV-2 and PMPV-1 appear to be most similar to JPV, TlmPV, BeiPV and Rodent PV. A schematic overview of the genome organization of these seven viruses is depicted in Fig. 3. An interesting feature shared by these viruses is the presence of additional genes between the F and G ORFs, encoding a small hydrophobic protein (‘SH’) and/or a transmembrane protein (‘TM’). While putative SH genes are present in a limited number of other paramyxoviruses (Mojiang henipavirus, Avian avulavirus 6, Mammalian rubulavirus 5, Mumps rubulavirus and Bat mumps rubulavirus), the TM gene, encoding a protein thought to be involved in cell-to-cell fusion, seems to be a unique feature of JPV, TlmPV, BeiPV and Rodent PV [21, 22]. In the PMPV-1 genome, both the SH and TM genes are present, encoding an 82 and a 256 amino acid protein, respectively. Conversely, the MMLPV-1 and MMLPV-2 genomes encode only the TM protein, setting them apart from all currently known paramyxoviruses. The TM proteins of MMLPV-1, MMLPV-2, PMPV-1, JPV, BeiPV, TlmPV and Rodent PV are between 218 and 275 amino acids long and share a similar structural organization, marked by the presence of a transmembrane domain between positions 56–81.

### Comparison of the G gene of MMLPV-1, MMLPV-2 and PMPV-1

**Fig. 4:**
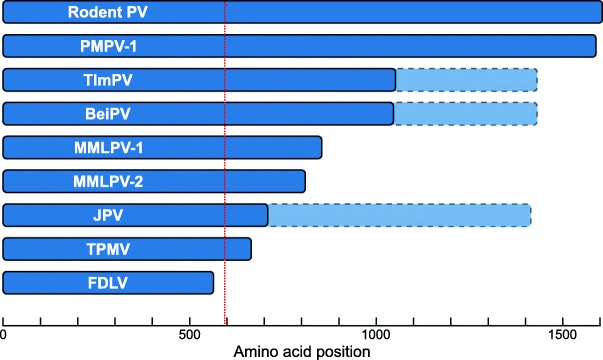
Comparison of the G ORFs of all ‘Jeilongviruses’. The G ORFs of the seven known ‘Jeilongviruses’ show a remarkable variation in size, and all of them are larger than those of other paramyxoviruses. The dashed red line marks the average size of a paramyxovirus G ORF. The G ORFs of Tupaia paramyxovirus (TPMV) and Fer-de-lance virus (FDLV) are included to illustrate both the exceptional size of the ‘Jeilongvirus’ G ORFs and their mutual variation, as these two viruses have the largest and smallest G ORFs of all non-‘Jeilongvirus’ paramyxoviruses, respectively. The light blue extensions indicate the complete size of the BeiPV, TlmPV and JPV G genes, including the X ORFs

An interesting genome feature of the paramyxoviruses described here is the size and nucleotide composition of their G genes. In members of the family *Paramyxoviridae*, the G gene encodes the larger of the two viral glycoproteins, which plays a role in the binding of target cells. This G protein, sometimes designated H (or HN) if the protein has hemagglutination (and neuraminidase) activity, is typically ~ 600 amino acids long and consists of a small luminal domain, a transmembrane region and a large ectodomain [23]. In the genomes of MMLPV-1, MMLPV-2, and PMPV-1, the G gene is much larger, encoding proteins that are, respectively, 854, 810, and 1589 amino acids long. Especially the PMPV-1 G protein is extraordinarily large, reaching ~ 2.5× the size of the average paramyxovirus G protein (Fig. 4).

**Fig. 5:**
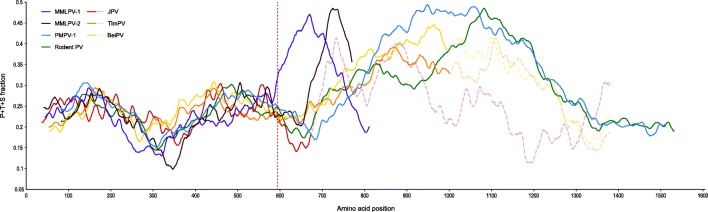
Sliding window overview of the amino acid composition of all Jeilongvirus G proteins. The used window size for each protein is 10% of the respective protein length. The dotted red line represents the average length of paramyxovirus G proteins. All Jeilongvirus G proteins are marked by the presence of an otherwise absent C-terminal region, marked by a high, yet variable abundance of P/T/S (up to 50%). In the genomes of JPV, BeiPV and TlmPV, the G gene is divided into two ORFs, G and X. A single mutation/insertion, however, suffices to join these two ORFs into a single ORF covering the entire G gene. The P/T/S fraction of the resulting hypothetical BeiPV and TlmPV G proteins (yellow/orange dashed lines) is similar to those of the Rodent PV and PMPV-1 G proteins. For JPV, which is more closely related to MMLPV-1/2 (Fig. 2, Additional file 1: Figure S1), this effect is less pronounced, although a narrow peak in P/T/S-abundance, similar to the MMLPV-2 peak, can be observed in the resulting hypothetical JPV G protein (dashed red line)

A number of observations can be made about these unusually large G proteins. Firstly, the exceptional size of these proteins seems to be completely attributable to the size of their respective ectodomains, as the combined length of the luminal and transmembrane domains does not exceed 70 amino acids for either one of these viruses. Secondly, although the G proteins of these three viruses are characterized by their large size, there are some notable dissimilarities between them. In contrast to the first ~ 600 amino acids of these proteins, which are markedly similar to the G proteins of other paramyxoviruses, the residual C-terminal region varies significantly not only in length but also in terms of its sequence, with no notable resemblance between the different viruses. Another interesting aspect of these G proteins is the amino acid composition of their C-terminal region. Despite the lack of apparent homology, the amino acid composition of the C-terminal region appears to follow a similar pattern for these three viruses, as this region is marked by the relative abundance of proline, threonine and, to a lesser extent, serine, with these amino acids constituting up to 45% of all amino acids in this region (Fig. 5). This enrichment of P/T/S is particularly striking in the case of PMPV-1, where the P/T/S-rich region spans more than 400 amino acids, to such an extent that it can even be observed on the nucleotide level, explaining our difficulties in amplifying this region (see above; Fig. 1).

## Discussion

In this article, we present three previously undescribed paramyxoviruses found in a bank vole from Slovenia and in a Mozambican Rungwe brush-furred rat. Using a combination of Illumina and Sanger sequencing, we determined the complete genome sequence of these three viruses. Sequence comparison of the viral genomes with known paramyxoviruses showed that each of these three viruses should be classified as a new species within the family *Paramyxoviridae*. This was further confirmed by phylogenetic inference using the concatenated protein sequence of all currently known (putative) paramyxovirus species, which showed that, while all three viruses cluster together with BeiPV, TlmPV, JPV and the recently discovered Rodent PV, they represent three separate, perfectly supported, lineages within the paramyxovirus family (Fig. 2, Additional file 1: Figure S1). BeiPV, TlmPV and JPV have been previously suggested to constitute a separate genus in the family *Paramyxoviridae*, clustering near the genera *Henipavirus* and *Morbillivirus*, and the yet unclassified Salem virus, Tupaia paramyxovirus, Nariva virus, Bank vole virus and Mossman virus [10, 24]. The viruses presented here provide additional support for the creation of this genus, which has been tentatively called ‘Jeilongvirus’, a conjunction of the names J-virus and Beilong virus.

PMPV-1 was discovered in a Slovenian bank vole. With a genome length of 20,148 nucleotides, it is the largest mammalian paramyxovirus reported to date. The exceptional size of this virus can partly be attributed to the presence of the SH and TM genes, which are absent in most other paramyxoviruses, but even more so to the large size of the G gene of this virus. Concurrently with this study, Alkhovsky and colleagues published an article describing the complete genome sequence of Bank vole virus, a new paramyxovirus species isolated from a Russian bank vole [8]. Interestingly, this virus was isolated from the same host species but shares little similarity with PMPV-1 (36% pairwise identity according to PASC) [19]. BaVV is most similar to Mossman virus, Nariva virus and Tupaia paramyxovirus and does not cluster with members of the putative genus ‘Jeilongvirus’. The complete genome of BaVV is 16,992 nucleotides long, more than 3 kb shorter than PMPV-1. It lacks the SH and TM genes and its G protein is much smaller than that of PMPV-1 (625 amino acids compared to 1589). As the G protein is involved in facilitating cell entry by interacting with specific proteins on the surface of target cells, this protein is most likely the predominant determinant of paramyxovirus host specificity and different paramyxovirus G proteins have probably evolved to allow optimal interaction with their respective host’s target cells [25]. The fact that this protein is so divergent between these two viruses, despite the shared host species, could indicate that they employ different entry receptors, although the identity of these receptors remains to be elucidated. It is also possible that bank voles are only an accidental host for PMPV-1 and that the virus has a different, yet to be determined natural host.

Perhaps even more intriguing than the fact that PMPV-1 and BaVV seem to share the same host species, is the observation that MMLPV-1 and MMLPV-2 were isolated from the same animal, potentially implying they share the same natural host. However, although some paramyxoviruses show a certain degree of host specificity, many species, especially members of the genera *Henipavirus* and *Rubulavirus*, have been found in multiple host species [26–28]. It is, therefore, possible that MMLPV-1 and/or MMLPV-2 have a natural host other than the Rungwe brush-furred rat, and that the co-infection observed here is merely a coincidental finding. Nonetheless, it is evident that infection with either one of these viruses does not impede a secondary infection, but an intriguing question that remains unanswered is whether these viruses compete or cooperate with each other in terms of host exploitation, or merely co-exist without significant interaction. Although the G proteins of these two viruses show a certain degree of similarity (Table 1), their C-terminal regions are strongly divergent. As mentioned above, the G protein acts as the cell attachment receptor and plays an important role in target cell binding and entry. Given the fact that MMLPV-1 and MMLPV-2 have markedly different G proteins, their evolution seems to have led them to utilize different receptors for cell attachment in the same host. Furthermore, because the G protein, besides the F protein, is one of the primary targets of the adaptive immune system in paramyxovirus infections, this difference may also have important implications for the host’s immune response against MMLPV-1 and MMLPV-2 [29]. However, whether these putative differences in virus-host interaction actually influence the interplay between these two viruses during co-infection remains to be established.

As mentioned above, perhaps the most interesting feature of PMPV-1, MMLPV-1 and MMLPV-2 are their G proteins. Comparable to other members of the putative genus ‘Jeilongvirus’, the size of these proteins strongly exceeds that of other paramyxovirus G/H/HN proteins (Fig. 4). While there is little sequence conservation, the C-terminal region of these proteins has a unique amino acid composition, marked by a relative abundance of P/T/S (Fig. 5). A single exception to this observation seems to be the G protein of JPV, which is only slightly larger than the average G/H/HN protein and shows no P/T/S enrichment [30]. Intriguingly, in the genome of JPV, as in those of BeiPV and TlmPV, the G gene is followed by an ORF, tentatively designated ‘ORF X’, that is absent in the genomes of all other paramyxoviruses. These X ORFs are considered part of the G gene, as they do not have their own gene start and stop signals, making it unlikely that they are transcribed independently. Concordantly, in the case of JPV, Jack and colleagues showed that the X ORF, which is located directly behind the G ORF and on the same frame, is only expressed in tandem with the G ORF, as part of a single 4.4 kb mRNA, confirming that the X ORF forms an integral part of the JPV G gene [31]. Whether the X ORF also gets translated, either independently from or conjointly with the G ORF, forming a ‘G-X’ fusion protein, or is simply the product of a recently acquired mutation, resulting in the formation of a stop codon within the G gene, remains to be determined. A number of arguments, however, seem to support the latter hypothesis. Firstly, at least for JPV, the X ORF does not seem to be expressed in virus-infected cells, neither separately nor as part of a G-X fusion protein [31]. Secondly, a single mutation (JPV) or insertion (BeiPV and TlmPV) suffices to ‘restore’ the G gene to a single ORF with a length similar to that of the G gene of PMPV-1 and Rodent PV. Thirdly, expression of these ‘restored’ G genes would result in proteins with amino acid-composition patterns similar to those of the PMPV-1 G protein (e.g. an abundance of P/T/S in the C-terminal half of the protein) (Fig. 5). Altogether, these findings suggest that, whereas MMLPV-1, MMLPV-2, PMPV-1 and Rodent PV still have a P/T/S-enriched ectodomain in their G protein, this feature has been (partially) lost in the case of BeiPV, TlmPV and JPV. P/T/S-enrichment is something that is not seen in the G/H/HN proteins of other paramyxoviruses, but it is a known characteristic of the attachment protein of members of the family *Pneumoviridae* (a former subfamily of the family *Paramyxoviridae*) [32]. In these viruses, the attachment G protein contains two ‘mucin-like’ domains that are rich in P/T/S, allowing for an extensive glycosylation of this protein [33]. Although its precise function is still unclear, the extensive glycosylation of this protein is thought to aid in shielding the protein from recognition by the host’s immune system. The presence of putative glycosylation sites that promote immune evasion hence provides a potential explanation for the unusual amino acid composition of the G protein of members of the genus ‘Jeilongvirus’. Further research is needed, however, to determine why this region varies so strongly between these seven related viruses, why BeiPV, TlmPV and JPV seem to have partially lost this P/T/S-rich region and why this particular region is absent altogether in the genomes of other members of the family *Paramyxoviridae*.

In addition to an unusually large G gene, the viruses presented here are also marked by the presence of additional ORFs (SH/TM) between the F and G genes. The first paramyxoviruses to be described with this particular genome organization were JPV and BeiPV, later followed by TlmPV. As previously mentioned, these viruses have been suggested to constitute a separate genus within the family *Paramyxoviridae* (genus ‘Jeilongvirus’), based on their unique characteristics and evolutionary distance from other paramyxoviruses. The newly characterized viruses described here form a substantial expansion of this putative genus, and their mutual similarities provide further justification for its establishment. Intriguingly, whilst the TM gene and large G gene seem to be conserved features of these viruses, the SH gene is absent in the genomes of MMLPV-1 and MMLPV-2. This gene encodes a small hydrophobic protein that, in the case of JPV, has been shown to downregulate TNF-α expression by interacting with the NFκB-pathway [34]. Why this gene is absent in the genomes of MMLPV-1 and MMLPV-2 remains to be determined, although the fact that these two viruses seem to share the same host may indicate that the absence of this gene is the result of host-specific adaptation.

## Conclusions

In conclusion, the current study presents the complete genome sequences of Mount Mabu Lophuromys paramyxovirus 1, Mount Mabu Lophuromys paramyxovirus 2 and Pohorje Myodes paramyxovirus 1, three new paramyxoviruses detected in rodents. The genome organization of these viruses (3’-N-P/V/C-M-F-SH-TM-G-L-5′) follows that of JPV, BeiPV, TlmPV and Rodent PV, except for the absence of the SH gene in the genomes of MMLPV-1 and MMLPV-2. Based on phylogenetic inference, these three viruses are thought to represent three new species within the family *Paramyxoviridae*, as part of the yet to be established genus ‘Jeilongvirus’.

## Methods

### Sample collection and Illumina sequencing of MMLPV-1 and MMLPV-2

The spleen and kidneys of an adult female Rungwe brush-furred rat, snap-trapped in July 2011 in an old banana plantation starting to be overgrown by Afromontane forest on Mount Mabu, Mozambique (coordinates: 16.3086S, 36.4245E), were collected and stored in RNAlater (QIAGEN Benelux, Venlo, The Netherlands). RNA was extracted from one of the kidneys according to the viral enrichment protocol S3, described in [35], using 25 U RNase ONE Ribonuclease (Promega Benelux, Leiden, The Netherlands) and 30 U Benzonase Nuclease (Sigma-Aldrich, St. Louis, MS, USA) for RNA digestion (90 min at 37 °C). Elution was performed twice by collecting and loading the same eluate on the column. Subsequently, the sample was quantitated using the RNA Quantifluor System (Promega) and an Agilent RNA 6000 Pico chip, loaded on a Bioanalyzer 2100 (Agilent Technologies, Santa Clara, CA, USA). Viral RNA was subjected to pre-amplification, using the Ovation RNA-Seq System V2 (NuGEN Technologies, San Carlos, CA, USA) for cDNA generation. The sequencing library was constructed with the Ovation UltraLow Library System V2 (NuGEN Technologies) and paired-end sequencing was performed on an Illumina NextSeq 500 (Illumina, Hayward, CA, USA).

### Sample collection and Illumina sequencing of PMPV-1

An adult male bank vole was trapped in July 2005 in a dense mixed-forest area surrounding a gorge stream on the Pohorje Massif, Slovenia (coordinates: 46.5126 N, 15.3386E). The bank vole was anaesthetized with isoflurane using the drop-jar method and subsequently euthanized by cardiac puncture, after which the internal organs were removed aseptically. Kidney and lung samples were pooled and homogenized in sterile PBS using a Minilys homogenizer (Bertin Technologies, Montigny-le-Bretonneux, France). The homogenate was passed through a 0.8 μm centrifugal filter with a hydrophilic polyvinylidene fluoride membrane (EMD Millipore, Burlington, MA, USA). Subsequently, virus particles were purified, based on the protocol by Stang and co-authors, by layering 10 ml of filtered supernatant onto 2 ml of 30% (wt/vol) sucrose-PBS and centrifuging at 30,000 rpm for 3 h in an SW41 rotor [36]. Total RNA was extracted from the resulting eluate using the QIAamp Viral RNA Mini kit (QIAGEN), according to the manufacturer’s instructions with the omission of carrier RNA, followed by enzymatic digestion of DNA using 2 U of TURBO DNase (Thermo Fisher Scientific, Waltham, MA, USA). The RNA extract was cleaned up and concentrated using the RNeasy MinElute Cleanup kit (QIAGEN). The Ribo-Zero Gold rRNA Removal Kit (Epidemiology)(Illumina) and the Dynabeads mRNA DIRECT Purification Kit (Thermo Fisher Scientific) were used to deplete rRNA and mRNA, according to the manufacturer’s instructions. After concentration with the RNeasy MinElute Cleanup kit (QIAGEN), the RNA was subjected to random amplification using the Complete Whole Transcriptome Amplification Kit WTA2 (Sigma-Aldrich). WTA2 products were purified using the MSB Spin PCRapace kit (Stratec, Birkenfeld, Germany). Sequencing libraries were prepared using the Nextera Library Prep Kit (Illumina) and paired-end sequenced on an Illumina NextSeq 500 system.

### Genome assembly and species confirmation

Illumina reads from both runs were trimmed and de novo assembled using CLC Genomics Workbench v10.1.1 (QIAGEN). A tBLASTx search of the resulting contigs against the virus database (taxid: 10239) identified two contigs for the Slovenian sample and five for the Mozambican sample that displayed significant similarity to paramyxoviruses. Mapping of the trimmed reads against these contigs showed that two of the Mozambican contigs had erroneous endings. Trimming of these erroneous bases and correct extension of the afflicted contigs allowed joining, using Seqman (v7.0.0), the five Mozambican contigs into two ~ 17 kb contigs, corresponding to two complete paramyxovirus genomes. The two contigs from the Slovenian sample (11.2 and 8.5 kb) corresponded to two halves of a paramyxovirus genome, but could not be joined into one contig. A BLASTn search of the de novo assembled contigs against a cytochrome b gene database was used to confirm the presumed identity of the two host animals. The sequences of the two hosts’ cytochrome b genes were submitted to GenBank under accession numbers MH197068 and MH197069.

### Genome finishing using sanger sequencing

To close the remaining gap in the sequence of the PMPV-1 genome, the OneStep RT-PCR Kit (QIAGEN) was used to generate a 1500 bp-amplicon spanning the entire gap length. The temperature for reverse transcription (RT) was increased to 60 °C to improve RT efficiency. Following purification using ExoSAP-IT (Affymetrix, High Wycombe, UK), the resulting PCR product was prepared for sequencing using the BigDye Terminator v3.1 Cycle Sequencing Kit (Applied Biosystems, Carlsbad, CA, USA). Sequencing was performed on an ABI Prism 3130xl Genetic Analyzer (Thermo Fisher Scientific). Sanger sequencing was also used to determine the sequence of the genome ends of PMPV-1. This was done by generating poly-A/T-tailed cDNA of both 5′ and 3′ ends using the 5′/3’ RACE kit 2nd generation (Roche, Mannheim, Germany). For the 3′ end, a poly-A-tail was added directly to the viral RNA prior to cDNA generation using the Poly(A) Polymerase Tailing Kit (Epicentre, Madison, WI, USA) according to the manufacturer’s instructions. This poly-A/T-tailed cDNA was used in combination with the OneStep RT-PCR Kit (QIAGEN) to amplify the genome ends and the resulting PCR products were prepared for Sanger sequencing as described above. All chromatogram files were inspected with Chromas (v2.6.2) before joining the obtained sequences with the Illumina contigs using Seqman (v7.0.0). All used primer sequences and PCR cycling conditions are available on request.

### Phylogenetic analysis

Amino acid sequences of the N, P, M, F, G and L open reading frames (ORF) of all recognized and putative paramyxovirus species for which the complete genome sequence is available in GenBank were concatenated and subsequently aligned using MAFFT (v7.123b) [37]. Four newly discovered fish paramyxoviruses were excluded from this analysis, as it is not always clear which of their ORFs corresponds to a P, M, F or G ORF. Following trimming with trimAL (1.2rev59), using the gappyout method, MEGA7 was used to visually inspect the resulting multiple sequence alignments [38, 39]. BEAST (v1.8.4), using default priors and assuming homochronous tips, was used to infer Bayesian phylogenetic trees, employing a WAG + Γ model to describe the amino acid substitution process [40–42]. Upon running the Markov chain Monte Carlo analyses until adequate sample sizes (ESS > 200) were obtained, TreeAnnotator (v2.5.4) was used to summarize a maximum clade credibility tree from the posterior tree distribution, using a 10% burn-in. FigTree (v1.4.3) was used for the visualization of the resulting tree. A secondary tree, incorporating the newly discovered fish paramyxoviruses but based only on the L ORF, was made analogously.

### Identity-based comparison with other paramyxoviruses

Multiple sequence alignments of the different proteins of all Jeilongviruses and a representative member of each paramyxovirus genus (including BaVV for the yet unclassified clade containing Mossman virus, Bank vole virus, Nariva virus and Tupaia paramyxovirus) were made using MAFFT (v7.123b) [37]. MEGA7 was used for pairwise distance analysis, calculating the number of differences between each sequence pair [39]. Sites containing gaps in the sequence alignment were omitted from the analysis.

### Jeilongvirus G ORF/protein nt/aa composition analysis

The nucleotide composition of the PMPV-1 G ORF (Fig. 1) was determined using a custom Python script that calculates content percentages for each of the different nucleotides using a sliding window size of 5% of the total gene length, incrementing over the sequence with steps of 1 nucleotide. A slightly modified version of this script was used to calculate the P/S/T contents of the different Jeilongvirus G proteins (Fig. 5), employing a sliding window size of 10% of each respective protein. The resulting graphs (Figs. 1 and 5) were smoothened by calculating, for each series of data points, a moving average curve with a period size of 10.

## Additional files

Additional file 1: **Figure S1.** Maximum clade credibility tree of 69 currently known paramyxovirus species. Expanded version of the tree provided in Fig. 2. The tree is based on Bayesian phylogenetic inference of the concatenated sequences of the N, P, M, F, G and L proteins of all 55 currently recognized paramyxovirus species, as well as 14 putative species that have not yet been classified (marked with ‘*’). Branch lengths are scaled and represent the number of amino acid substitutions per site. Numbers at the different nodes indicate the posterior support for each cluster. (DOCX 246 kb)

Additional file 2: **Figure S2.** Maximum clade credibility tree of all currently known paramyxovirus species. The tree is based on Bayesian phylogenetic inference of the L proteins of all 55 currently recognized paramyxovirus species, as well as 18 putative species that have not yet been classified (marked with ‘*’). Branch lengths are scaled and represent the number of amino acid substitutions per site. Numbers at the different nodes indicate the posterior support for each cluster. (DOCX 289 kb)

Additional file 3: **Table S1.** List of GenBank accession numbers of all viral sequences used, ordered by genus. A single representative sequence was used for each (putative) paramyxovirus species. Viruses marked with an ‘*’ represent putative species that have not yet been recognized by ICTV. (DOCX 24 kb)
