# Supplementary material for: Discovery and genome characterization of three new Jeilongviruses, a lineage of paramyxoviruses characterized by their unique membrane proteins
Source: BMC Genomics. 2018 Aug 16;19:617. doi: 10.1186/s12864-018-4995-0 (PMC6097224; doi:10.1186/s12864-018-4995-0)
Supplement: Supplementary file 1 — Figure S1. Maximum clade credibility tree of 69 currently known paramyxovirus species. Expanded version of the tree provided in Fig. 2. The tree is based on Bayesian phylogenetic inference of the concatenated sequences of the N, P, M, F, G and L proteins of all 55 currently recognized paramyxovirus species, as well as 14 putative species that have not yet been classified (marked with ‘*’). Branch lengths are scaled and represent the number of amino acid substitutions per site. Numbers at the different nodes indicate the posterior support for each cluster. (DOCX 246 kb) [file 12864_2018_4995_MOESM1_ESM.docx]

**
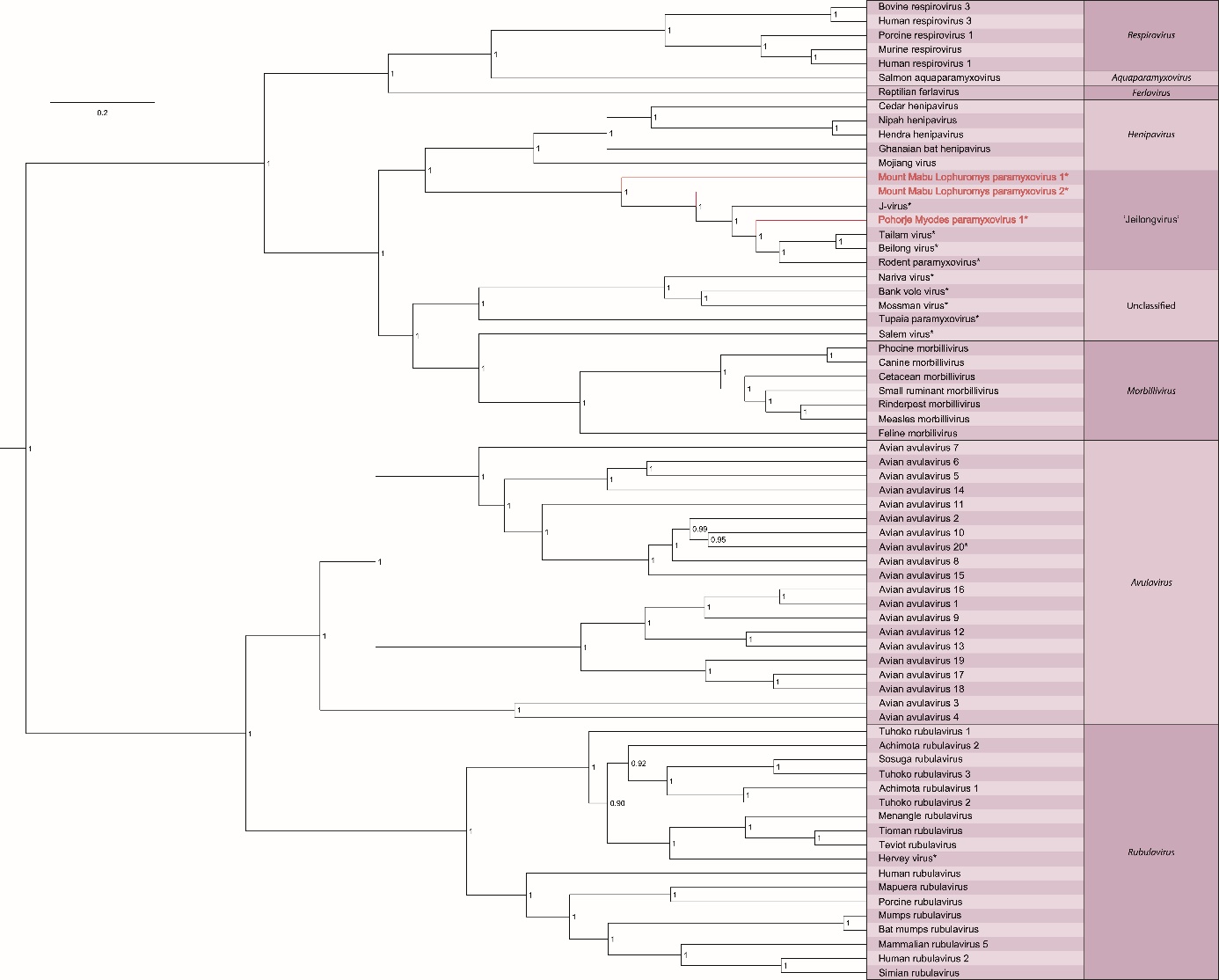
**

**Fig. A1: Maximum clade credibility tree of 69 currently known paramyxovirus species.** Expanded version of the tree provided in Figure 2. The tree is based on Bayesian phylogenetic inference of the concatenated sequences of the N, P, M, F, G and L proteins of all 55 currently recognized paramyxovirus species, as well as 14 putative species that have not yet been classified (marked with ‘*’). Branch lengths are scaled and represent the number of amino acid substitutions per site. Numbers at the different nodes indicate the posterior support for each cluster.
