# Supplementary material for: Discovery and genome characterization of three new Jeilongviruses, a lineage of paramyxoviruses characterized by their unique membrane proteins
Source: BMC Genomics. 2018 Aug 16;19:617. doi: 10.1186/s12864-018-4995-0 (PMC6097224; doi:10.1186/s12864-018-4995-0)
Supplement: Supplementary file 2 — Figure S2. Maximum clade credibility tree of all currently known paramyxovirus species. The tree is based on Bayesian phylogenetic inference of the L proteins of all 55 currently recognized paramyxovirus species, as well as 18 putative species that have not yet been classified (marked with ‘*’). Branch lengths are scaled and represent the number of amino acid substitutions per site. Numbers at the different nodes indicate the posterior support for each cluster. (DOCX 289 kb) [file 12864_2018_4995_MOESM2_ESM.docx]

**
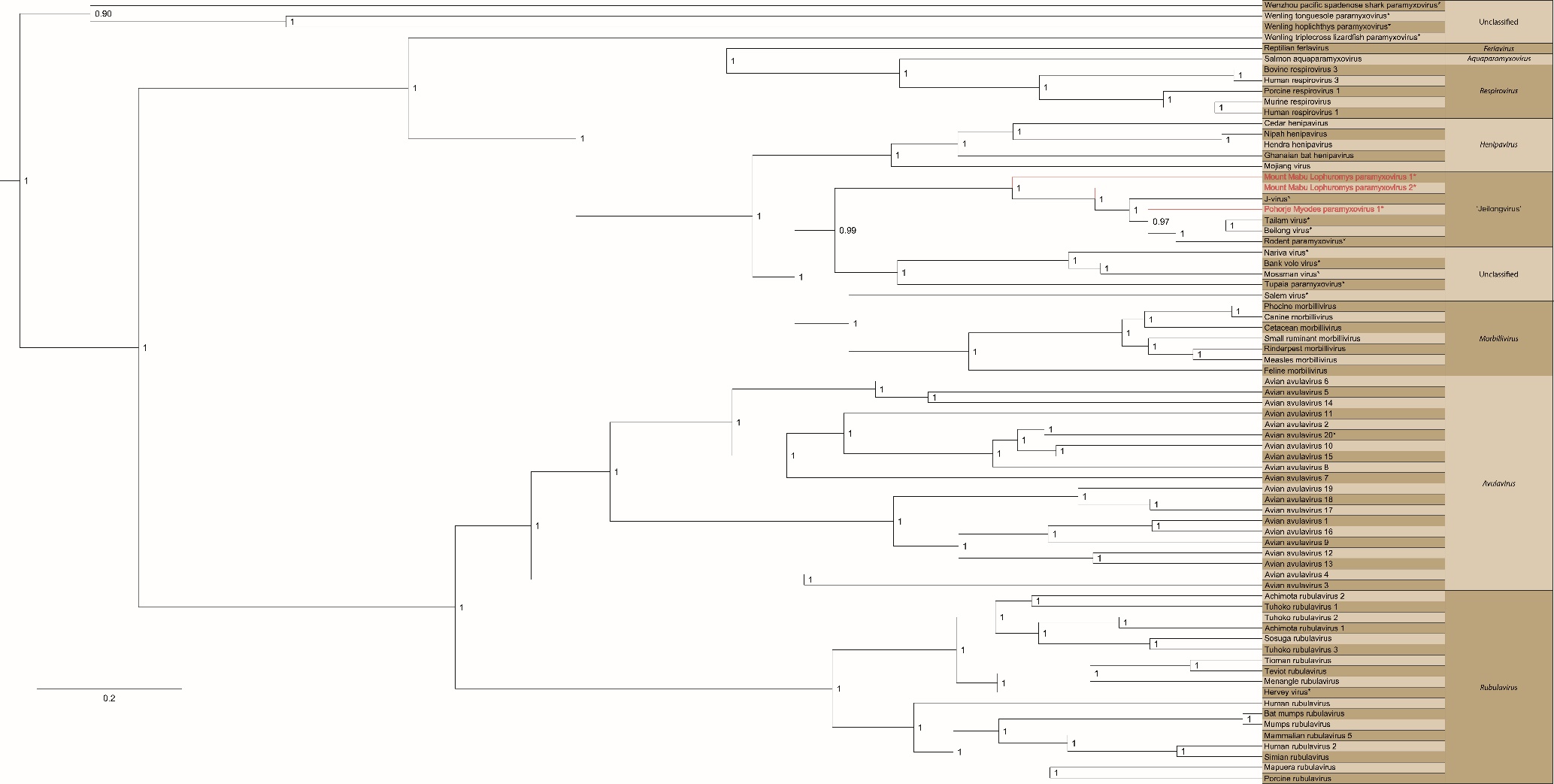
**

**Fig. A2: Maximum clade credibility tree of all currently known paramyxovirus species.** The tree is based on Bayesian phylogenetic inference of the L proteins of all 55 currently recognized paramyxovirus species, as well as 18 putative species that have not yet been classified (marked with ‘*’). Branch lengths are scaled and represent the number of amino acid substitutions per site. Numbers at the different nodes indicate the posterior support for each cluster.
