# Supplementary material for: Discovery and genome characterization of three new Jeilongviruses, a lineage of paramyxoviruses characterized by their unique membrane proteins
Source: BMC Genomics. 2018 Aug 16;19:617. doi: 10.1186/s12864-018-4995-0 (PMC6097224; doi:10.1186/s12864-018-4995-0)
Supplement: Supplementary file 3 — Table S1. List of GenBank accession numbers of all viral sequences used, ordered by genus. A single representative sequence was used for each (putative) paramyxovirus species. Viruses marked with an ‘*’ represent putative species that have not yet been recognized by ICTV. (DOCX 24 kb) [file 12864_2018_4995_MOESM3_ESM.docx]

| Genus | virus (1 per species) | Accession number |
| --- | --- | --- |
| *Aquaparamyxovirus* | Salmon aquaparamyxovirus | EU156171.1 |
| *Avulavirus* | Avian avulavirus 1 | AF309418.1 |
|  | Avian avulavirus 2 | HM159993.1 |
|  | Avian avulavirus 3 | EU782025.1 |
|  | Avian avulavirus 4 | JX987283.1 |
|  | Avian avulavirus 5 | GU206351.1 |
|  | Avian avulavirus 6 | AY029299.1 |
|  | Avian avulavirus 7 | FJ231524.1 |
|  | Avian avulavirus 8 | FJ619036.1 |
|  | Avian avulavirus 9 | EU910942.1 |
|  | Avian avulavirus 10 | HM147142.3 |
|  | Avian avulavirus 11 | JQ886184.1 |
|  | Avian avulavirus 12 | KC333050.1 |
|  | Avian avulavirus 13 | LC041132.1 |
|  | Avian avulavirus 14 | KX258200.1 |
|  | Avian avulavirus 15 | KX932454.2 |
|  | Avian avulavirus 16 | KY511044.1 |
|  | Avian avulavirus 17 | KY452442.1 |
|  | Avian avulavirus 18 | KY452443.1 |
|  | Avian avulavirus 19 | KY452444.1 |
|  | Avian avulavirus 20* | MF033136.1 |
| *Ferlavirus* | Reptilian ferlavirus | AY141760.2 |
| *Henipavirus* | Cedar henipavirus | JQ001776.1 |
|  | Ghanaian bat henipavirus | HQ660129.1 |
|  | Hendra henipavirus | AF017149.3 |
|  | Mojiang henipavirus | KF278639.1 |
|  | Nipah henipavirus | AF212302.2 |
| ‘Jeilongvirus’ | Beilong virus* | KX940961.1 |
|  | J-virus* | AY900001.1 |
|  | Mount Mabu Lophuromys paramyxovirus 1* | MG573140 |
|  | Mount Mabu Lophuromys paramyxovirus 2* | MG573141 |
|  | Rodent paramyxovirus* | KY370098.1 |
|  | Pohorje Myodes paramyxovirus 1* | MG516455 |
|  | Tailam virus* | JN689227.1 |
| *Morbillivirus* | Canine morbillivirus | AF014953.1 |
|  | Cetacean morbillivirus | AJ608288.1 |
|  | Feline morbillivirus | AB924120.1 |
|  | Measles morbillivirus | AB016162.1 |
|  | Phocine morbillivirus | KC802221.1 |
|  | Rinderpest morbillivirus | X98291.3 |
|  | Small ruminant morbillivirus | AJ849636.2 |
| *Respirovirus* | Bovine respirovirus 3 | AF178654.1 |
|  | Human respirovirus 1 | AF457102.1 |
|  | Human respirovirus 3 | AB012132.1 |
|  | Murine respirovirus | AB005795.1 |
|  | Porcine respirovirus 1 | JX857409.1 |
| *Rubulavirus* | Achimota rubulavirus 1 | JX051319.1 |
|  | Achimota rubulavirus 2 | JX051320.1 |
|  | Bat mumps rubulavirus | HQ660095.1 |
|  | Hervey virus* | KU672593.1 |
|  | Human rubulavirus 2 | X57559.1 |
|  | Human rubulavirus 4 | AB543336.1 |
|  | Mammalian rubulavirus 5 | AF052755.1 |
|  | Mapuera rubulavirus | EF095490.1 |
|  | Menangle rubulavirus | AF326114.2 |
|  | Mumps rubulavirus | AB040874.1 |
|  | Porcine rubulavirus | BK005918.1 |
|  | Simian rubulavirus | X64275.1 |
|  | Sosuga rubulavirus | KF774436.1 |
|  | Teviot rubulavirus | KP271123.1 |
|  | Tioman rubulavirus | AF298895.2 |
|  | Tuhoko rubulavirus 1 | GU128080.1 |
|  | Tuhoko rubulavirus 2 | GU128081.1 |
|  | Tuhoko rubulavirus 3 | GU128082.1 |
| Unclassified | Bank vole virus* | MF943130.1 |
|  | Mossman virus* | AY286409.1 |
|  | Nariva virus* | FJ362497.2 |
|  | Salem virus* | JQ697837.1 |
|  | Tupaia paramyxovirus* | AF079780.2 |
|  | Wenling hoplichthys paramyxovirus* | MG600062.1 |
|  | Wenling tonguesole paramyxovirus* | MG600059.1 |
|  | Wenling triplecross lizardfish paramyxovirus* | MG600058.1 |
|  | Wenzhou pacific spadenose shark paramyxovirus* | MG600057.1 |

**Table A1**

List of GenBank accession numbers of all viral sequences used, ordered by genus. A single representative sequence was used for each (putative) paramyxovirus species. Viruses marked with an ‘*’ represent putative species that have not yet been recognized by ICTV.
